# Accuracy of large language model transcription of simulated physician-patient verbal interactions

**DOI:** 10.1186/s12911-026-03414-3

**Published:** 2026-03-10

**Authors:** Eric J. Nolan, Harry B. Burke

**Affiliations:** 1 Division of Hospital Medicine, Department of Internal Medicine, College of Medicine, The Ohio State University Wexner Medical Center, M112 Starling Loving Hall, 320 W. 10th Ave, Columbus, OH 43210 USA; 2 George Washington University School of Medicine, Washington, D.C USA

**Keywords:** Large language models (LLMs), Transcription, Clinical notes, Generative artificial Intelligence, Physician patient interaction, Ambient scribes

## Abstract

**Background:**

Large language models (LLM) are increasingly used in clinical medicine for tasks such as automated note generation. However, LLM-generated notes remain vulnerable to transcription errors, raising concerns about their reliability in clinical practice. We analyzed the types and rates of LLM mis-transcription errors (deletions, substitutions, and additions) and LLM mis-attribution errors (assigning dialogue to an incorrect speaker) in transcripts generated by a single LLM and tested whether error rates differed by speaker role and speaker sex. We also examined plausible sources of LLM-related error, including overlapping speech and speaking turn taking, and hypothesized that higher-quality audio would be associated with fewer transcription errors.

**Methods:**

In this retrospective single center study, an LLM (NotebookLM) generated speaker-labeled transcripts from audio recordings of twelve standardized-patient (SP) medical student encounters involving three SPs in a single simulated clinical scenario. Six encounters were re-recorded with higher-fidelity audio (HFA) to evaluate the effect of recording quality on errors. LLM-generated transcripts were compared with gold-standard transcripts. Outcomes included target word errors (substitutions and deletions), insertions, turn-taking errors, mis-attributed speaker word errors, semantic errors (errors that changed the meaning of a word or phrase), medical terminology errors, speaking turns, and overlapping speech.

**Results:**

Interactions averaged 2,226 ± 252 words and the mean transcription error frequencies were 73 ± 26 target word errors (3.3% of target words), 22 ± 13 substitutions (1.0%), 51 ± 22 deletions (2.3%), and 9 ± 4 insertions (0.4%). There were 19 ± 5 semantic word/phrase errors, of which 8 ± 4 were due to medical terminology errors. For speaker attribution accuracy, there were 15 ± 12 turn-taking errors (7.3% of all speaking turns), 5 ± 6 semantic turn-taking errors (errors that altered meaning), and 48 ± 39 mis-attributed-speaker word errors. Overlapping speech accounted for 19.1% of total word errors and 16.3% of mis-attributed speaker word errors. Speaking turns were correlated with target word errors and insertions (*r* = 0.41), turn-taking errors (*r* = 0.62), and mis-attributed speaker word errors (*r* = 0.51). HFA recordings reduced but did not eliminate the errors.

**Conclusions:**

In this single simulated clinical scenario involving three SPs and 12 SP–student interactions, overlapping speech, turn-taking, medical terminology, and audio fidelity were frequent contributors to transcription errors with NotebookLM. Though LLM transcription errors were modest, even small numbers of errors can have a meaningful impact on documentation. These findings suggest that caution is warranted when relying on LLMs for fully autonomous clinical note generation. LLMs may be most appropriately used in a supportive role, such as assisting clinicians in reviewing and improving physician-authored documentation, rather than replacing clinician involvement in the documentation process.

**Graphical Abstract:**

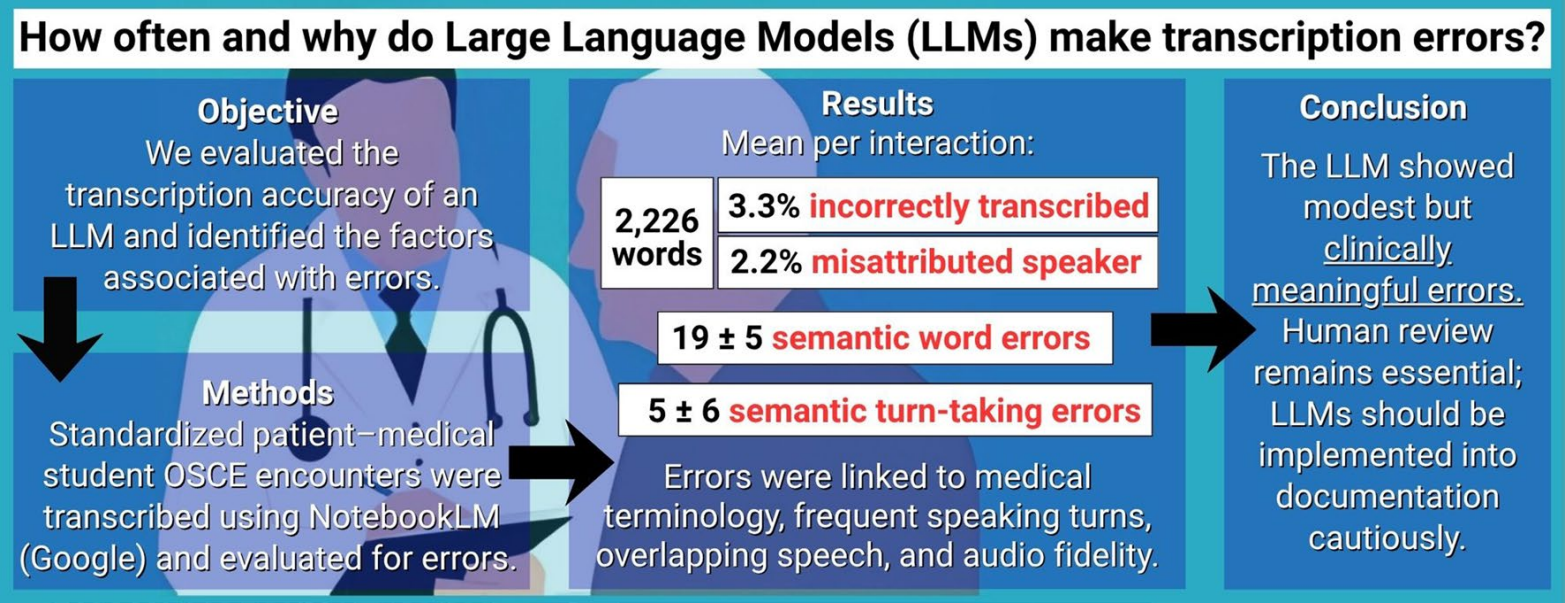

**Supplementary Information:**

The online version contains supplementary material available at 10.1186/s12911-026-03414-3.

## Background

Large language models (LLM) are being integrated into medical practice. Current applications include clinical information retrieval from the electronic medical record [1, 2], note review [3, 4], and patient education [5–7]. Increasingly, LLMs are also being used for ambient note generation, serving as virtual scribes that capture verbal physician–patient interactions (PPIs) and generate structured clinical documentation [8–13].

Accurate clinical documentation requires the reliable capture of the spoken interaction, yet prior studies have reported mis-transcription error rates ranging from 8.9% to 48% [14–18] and mis-attributed speaker word error rates of 1.8–13.9% [19]. This variability raises important concerns about the reliability of LLMs in clinical practice.

We are interested in quantifying the types and rates of LLM transcription errors. To provide a known gold standard and to control for setting, we used verbal standardized-patient (SP) student (performing as the physician) interactions.

After piloting several LLMs, we selected NotebookLM to generate transcripts. We analyzed the types and rates of LLM mis-transcription errors (deletions, substitutions, and additions) and mis-attribution errors (assigning dialogue to an incorrect speaker), and we tested whether error rates differed by speaker role and speaker sex. We also examined plausible sources of LLM-related error in the NotebookLM-generated transcripts, including overlapping speech and speaking turn taking, and hypothesized that higher-quality audio would be associated with fewer transcription errors.

## Methods

This single-institution retrospective study analyzed the audio recordings of SP interactions with third-year medical students. In each interaction, students obtained a focused medical history, performed a physical examination, and provided a diagnosis and treatment.

SPs were trained on the validated clinical case and given specific instructions regarding how to embody the case. Eight SPs participated in 52 recordings. To ensure an adequate pool of eligible interactions, we selected the three SPs with the highest number of recorded interactions. These SPs are designated as SP1, SP2, and SP3. From these SPs, we randomly selected four interactions per SP, yielding twelve verbal encounters; six of which were male students and six were female students.

A physician listened to and manually transcribed each interaction without automatic transcription tools to create gold-standard transcripts. The transcriber listened to each recording at least three times to ensure completeness and accuracy of the transcript. A second physician checked the results. During each interaction, an announcer provided three brief scripted prompts (start, 5-minute warning, and end; total of 19 words), which were manually removed from the transcripts prior to analysis.

Before defining study outcomes, we conducted a brief pilot of several LLMs to select a transcription platform based on transcription accuracy and speaker-attribution performance. The comparison was exploratory and driven by accuracy, cost, and availability at the time of the study. Google Cloud Speech-to-Text (Google, Mountain View, CA) was excluded because it was unable to provide speaker labeling for longer audio files and had a high error rate. At the time of the study, ChatGPT (OpenAI, San Francisco, CA) was unable to generate transcripts from uploaded audio files.

Otter.ai (AISense Inc., Los Altos, CA), AssemblyAI (AssemblyAI Inc., San Francisco, CA), and NotebookLM (Google, Mountain View, CA) were each piloted on the same two SP–student interactions. Performance was assessed by comparison with the reference transcripts, focusing on two metrics: word error rate (sum of substitutions, deletions, and insertions) and misattributed speaker word errors (Supplemental Table 1). Across the two interactions with a mean 2416 target words, NotebookLM demonstrated substantially lower overall word error rates (96 total word errors; 4.0% of target words) compared with AssemblyAI (252; 10.4%) and Otter.ai (448; 18.5%). AssemblyAI demonstrated the lowest rate of misattributed speaker word errors (34 total misattributed speaker word errors; 1.4%), compared with NotebookLM (86; 3.6%) and Otter.ai (216; 8.9%). NotebookLM was selected because its markedly lower overall word error rate was judged to be most critical for accurate analyses, despite AssemblyAI’s modest advantage in speaker attribution.

**Fig. 1:**
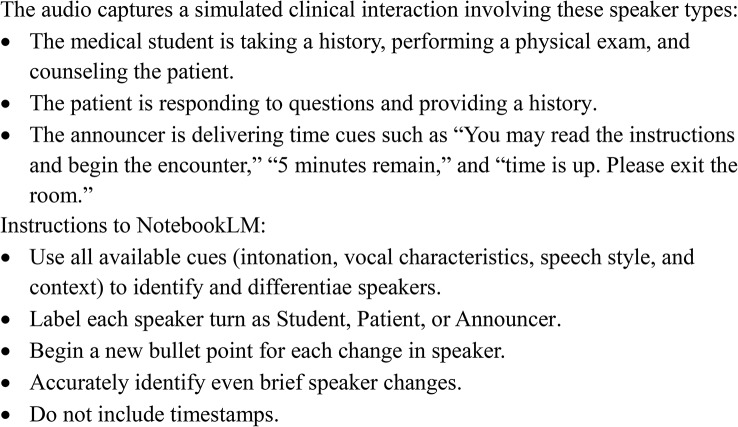
Prompt to NotebookLM

NotebookLM was accessed through its standard web-based interface. Each audio file was uploaded into a new NotebookLM session and transcribed using the same prompt (Fig. 1). Transcriptions were generated between May and June 2025 when the system was powered by Gemini 2.5 Flash. There were no changes to the system during this time interval, and we did not make any setting changes. Transcripts were produced as speaker-labeled lists and imported into Microsoft Word for analysis.

The audiovisual interactions were recorded by SimCapture (Laerdal Medical, Stavanger, Norway). The recordings were downloaded in .m4v format. We removed the student names from the metadata. The audio recordings were not altered, muted, clipped, or otherwise modified. Videos were discarded, and the audio was extracted using Audacity (Muse Group, Limassol, Cyprus) and saved as .wav files.

The (original-fidelity audio, OFA) recordings took place in carpeted 8 × 10 feet exam rooms with an 8-ft ceiling and a centrally mounted Parlé TCM-1 ceiling microphone (Biamp, Beaverton, Oregon, USA) using default settings. To evaluate the impact of audio quality, six of the OFAs were re-recorded using the gold-standard transcripts to generate higher-fidelity audio (HFA). For the HFA recordings, two individuals, matched by sex, role-played either the SP or the student. The re-enacted HFA interactions recordings took place in a quiet 8 × 10-ft hardwood room with an 8-ft ceiling. Speakers were 2–4 ft from a standard 39 g microphone (Noxgear, Worthington, OH, USA). The microphone was on its default settings with background noise reduction. Overlapping speech was not reproduced. Recordings were transcribed using the same NotebookLM protocol and compared with the OFA transcripts.

Terminology was defined as follows. “Target words” were words spoken by the SP or the student. “Mis-transcriptions” were LLM’s substitutions, deletions, and insertions of target words. “Substitutions” were words that were substituted for target words. “Deletions” were target words that were removed from the transcription. “Insertions” were words that were added to the transcription but were not target words. “Target word errors” were LLM substitutions and deletions words, they do not include insertions because they were not target words. “Total word errors” were target word errors and insertions. “Semantic word/phrase errors” were LLM mis-transcription (i.e., substitutions, deletions, or insertions) of words or phrases that altered an intended meaning. “Medical terminology errors” were LLM mis-transcriptions (i.e., substitutions, deletions, or insertions) of medical words unfamiliar to laypersons. If the error changed the meaning, it was also a semantic error. “Mis-attribution errors,” were when the LLM mis-attributed a speaker, identifying the wrong speaker in the dyad. Mis-attribution errors also counted as “turn-taking errors” and “mis-attributed speaker word errors.” “Speaking turns” were transitions from one speaker to the other speaker in the dyad. “Turn-taking errors” were when the LLM incorrectly marked a continued utterance as a new turn or failed to recognize a turn had occurred. Turn-taking errors can contain multiple words. If the turn-taking error changed intended meaning, it was a “semantic turn-taking error.” Mis-attributed speaker word errors were the total number of mis-attributed words. “Overlapping speech,” occurs when both the SP and the student spoke simultaneously. Errors associated with overlapping speech occur when words were mis-transcribed or mis-attributed during overlapping speech. Primary outcomes were word error rates. Secondary outcomes were errors by sex, speaker, and audio fidelity.

Percentages of target word errors, insertions, and mis-attributed speaker word errors were of the total target words, while percentages of turn-taking errors were of the total speaking turns. Results were reported as mean ± standard deviation in text and as mean (SD) in tables unless otherwise noted. Errors attributable to audio fidelity in the OFA interactions were estimated by adjusting for errors associated with overlapping speech and errors in HFA recordings. Total word errors and mis-attributed speaker word errors associated with overlapping speech were first subtracted from the total error counts in OFA interactions. The corresponding error counts in the HFA interactions were then subtracted to isolate the portion of errors attributable to audio fidelity.

The Pearson correlation coefficient (*r*) was used to assess the interactions and to evaluate relationships among word counts, speaking turns, instances of overlapping speech, and transcription error rates. Total target words, overlapping speech, speaking turns, and transcription errors were compared using the Student’s t-test with significance set at p-value ≤ 0.05. This study was reviewed and determined exempt by the Institutional Review Board.

## Results

On average, interactions lasted 14.3 ± 0.7 min and contained 2,226 ± 252 words (Table 1). The LLM produced a mean of 73 ± 26 target word errors (3.3% of all target words), comprising 22 ± 13 substitutions (1.0%) and 51 ± 22 deletions (2.3%). Insertions were less frequent (9 ± 4; 0.4%), and semantic word/phrase errors averaged 19 ± 5 per interaction.

**Table 1:** Comparison of total target words and LLM errors by standardized patient (SP) and by student. Percentages are out of total target words

	Student		Target Word Errors			Insertion errors	Semantic word/phrase errors (may span multiple words)
		Total target words	Total target word errors	Substitution errors	Deletion errors		
SP1	1	2139	112, 5.2%	56, 2.6%	56, 2.6%	15, 0.7%	27
	2	2693	61, 2.3%	18, 0.7%	43, 1.6%	4, 0.1%	22
	3	2326	102, 4.4%	24, 1.0%	78, 3.4%	5, 0.2%	22
	4	2006	98, 4.9%	21, 1.0%	77, 3.8%	6, 0.3%	26
SP1 mean (SD) %	1–4	2291 (298)	93 (22), 4.1%	30 (18), 1.3%	64 (17), 2.8%	8 (5), 0.3%	24 (3)
SP2	5	2137	63, 3.0%	22, 1.0%	41, 1.9%	14, 0.7%	22
	6	1912	70, 3.7%	36, 1.9%	34, 1.8%	14, 0.7%	16
	7	2563	32, 1.2%	12, 0.5%	20, 0.8%	10, 0.4%	10
	8	1973	30, 1.5%	18, 0.9%	12, 0.6%	7, 0.4%	16
SP2 mean (SD) %	5–8	2146 (294)	49 (21), 2.3%	22 (10), 1.0%	27 (13), 1.3%	11 (3), 0.5%	16 (5)
SP3	9	2284	67, 2.9%	17, 0.7%	50, 2.2%	9, 0.4%	17
	10	2041	63, 3.1%	8, 0.4%	55, 2.7%	5, 0.2%	15
	11	2515	82, 3.3%	10, 0.4%	72, 2.9%	12, 0.5%	14
	12	2128	91, 4.3%	16, 0.8%	75, 3.5%	11, 0.5%	20
SP3 mean (SD) %	9–12	2242 (208)	76 (13), 3.4%	13 (4), 0.6%	63 (12), 2.8%	9 (3), 0.4%	17 (3)
Overall mean (SD) %	1–12	2226 (252)	73 (26) 3.3%	22 (13), 1.0%	51 (22), 2.3%	9 (4), 0.4%	19 (5)

When comparing SP groups (Table 1), the total target word counts were similar across SPs, however, their error rates differed. SP2’s interactions had the lowest target word errors (49 ± 21, 2.3% vs. SP1: 93 ± 22, 4.1%; SP3: 76 ± 13, 3.4%) and semantic word/phrase errors (16 ± 5 vs. SP1: 24 ± 3; SP3: 17 ± 3). SP1’s interactions had the highest target word errors and semantic word/phrase errors. Insertions did not vary substantially.

Analysis of target words and errors by speaker (Table 2) demonstrated that students spoke significantly more words than SPs (1,660 ± 267 vs. 566 ± 166, respectively, *p* < 0.001). Despite this, SPs’ speech contained more target word errors (41 ± 21, 7.2%) than student speech (32 ± 14, 1.9%), while insertion and semantic word/phrase error rates did not differ meaningfully between groups. Within SP groups, SP2 spoke significantly fewer words (404 ± 31) than SP1 (624 ± 95, p value 0.019) and SP3 (670 ± 185, p value 0.029). SP2 also exhibited a lower target word error rate (17 ± 9; 4.2%) compared with SP1 (54 ± 15; 8.7%) and SP3 (51 ± 13; 7.6%), driven primarily by fewer deletions (SP2: 2.0% vs. SP1: 7.1% and SP3: 6.4%). Semantic word/phrase errors were likewise reduced in SP2’s interactions (6 ± 3 vs. SP1: 13 ± 2; SP3: 12 ± 3). In contrast, student dialogue was consistent across SP groups, with similar word counts and less variability in target word errors (1.6–2.3%) than amongst SP speakers (4.2–8.7%). The exception was students’ semantic word/phrase errors, which were lower in interactions with SP3’s interactions (4 ± 1) than in those with SP1 (12 ± 4) or SP2 (10 ± 2).

**Table 2:** Comparison of target words and LLM errors by speaker. Percentages are out of total target words

	Standardized Patient (SP) Speaker						Student Speaker					
		Target Word Errors						Target Word Errors				
	Target words	Total target word errors	Substi-tution errors	Deletion errors	Insertion errors	Semantic word/ phrase errors (may span multiple words)	Target words	Total target word errors	Substi- tution errors	Deletion errors	Insertion errors	Semantic word/ phrase errors (may span multiple words)
SP1 mean (SD) %	624 (95)*	54 (15) 8.7%	10 (6) 1.6%	44 (16) 7.1%	3 (1), 0.5%	13 (2)	1667 (244)*	39 (16) 2.3%	20 (12) 1.2%	20 (5) 1.2%	5 (4) 0.3%	12 (4)
SP2 mean (SD) %	404 (31)*	17 (9) 4.2%	9 (5) 2.2%	8 (5) 2.0%	6 (2) 1.5%	6 (3)	1743 (300)*	32 (14) 1.8%	13 (6) 0.7%	19 (11) 1.1%	6 (3) 0.3%	10 (2)
SP3 mean (SD) %	670 (185)*	51 (13) 7.6%	9 (3) 1.3%	43 (12) 6.4%	4 (3) 0.6%	12 (3)	1572 (269)*	25 (12) 1.6%	4 (2) 0.3%	20 (12) 1.3%	5 (2) 0.3%	4 (1)
Overall mean (SD) %	566 (166)	41 (21) 7.2%	9 (4) 1.6%	31 (21) 5.5%	4 (2) 0.7%	10 (4)	1660 (267)	32 (14) 1.9%	12 (10) 0.7%	20 (9) 1.2%	5 (3) 0.3%	9 (4)

SP mean target words vs. student mean target words (*N* = 12 per group): p value < 0.001

SP mean target words (*N* = 4 per group): SP1 vs. SP2, p value 0.019; SP1 vs. SP3, NS; SP2 vs. SP3, p value 0.029

In male SP-female student and male-SP male student dyads (Table 3), the SPs produced similar target word counts when paired with female students (583 ± 150) and male students (549 ± 194), with comparable target word error rates (44 ± 20, 7.5% vs. 37 ± 23, 6.7%). Female and male students also produced similar target word rates (1643 ± 270 and 1678 ± 289, respectively), and comparable error rates (27 ± 9, 1.6% and 37 ± 17, 2.2%).

**Table 3:** Comparison of LLM errors by speaker sex. Percentages are out of total target words

	Standardized Patient (SP) Speaker						Student Speaker					
		Target word Errors			Insertion errors	Semantic word/phrase errors (may span multiple words)		Target Word Errors			Insertion errors	Semantic word/phrase errors (may span multiple words)
	Target words	Total target word errors	Substi-tution errors	Deletion errors			Target words	Total target word errors	Substi-tution errors	Deletion errors		
Male SP-Female student mean (SD) %)	583 (150)	44 (20) 7.5%	8 (2) 1.4%	36 (20) 6.2%	3 (2), 0.5%	12 (4)	1643 (270)	27 (9) 1.6%	7 (4) 0.4%	19 (10) 1.2%	4 (2), 0.2%	7 (4)
Male SP- Male student mean (SD) %)	549 (194)	37 (23) 6.7%	11 (6) 2.0%	27 (22) 4.9%	5 (2), 0.9%)	9 (4)	1678 (289)	37 (17) 2.2%	17 (11) 1.0%	20 (9) 1.2%	6 (3) 0.4%	10 (4)

Speaking turns, turn-taking errors and mis-attributed speaker word errors are shown in Table 4. On average, interactions contained 206 ± 41 speaking turns, 15 ± 13 turn-taking errors (7.3% of turns), 48 ± 40 mis-attributed speaker word errors (2.2% of target words), and 5 ± 6 semantic turn-taking errors. SP1 had significantly more speaking turns (249 ± 18) than SP2 (168 ± 37, *p* = 0.007) and SP3 (SP3 202 ± 12, *p* = 0.006). SP1 also had the most turn-taking errors (26 ± 16, 10.5%) compared to SP2 (10 ± 9, 6.0%,) and SP3 (10 ± 5, 5.1%), as well as the most mis-attributed speaker word errors (73 ± 47, 3.2%) compared to SP2 (42 ± 43, 2.0%) and SP3 (29 ± 19, 1.3%).

**Table 4:** Comparison of LLM turn-taking errors and mis-attributed speaker word errors. Percent of total turn-taking errors is out of speaking turns; percent of mis-attributed speaker word errors are out of total target words

	Student	Speaking turns	Turn-taking errors	Mis-attributed speaker word errors
SP1	1	255	47, 18.4%	141, 6.6%
	2	255	9, 3.5%	31, 1.2%
	3	263	23, 8.7%	58, 2.5%
	4	222	23, 10.4%	62, 3.1%
SP1 mean (SD), %	1–4	249 (18)*	26 (16), 10.5%	73 (47), 3.2%
SP2	5	139	7, 5.0%	43, 2.0%
	6	143	7, 4.9%	21, 1.1%
	7	219	22, 10.0%	102, 4.0%
	8	169	2, 1.2%	3, 0.2%
SP 2 mean (SD) %	5–8	168 (37)*	10 (9), 6.0%	42 (43), 2.0%
SP3	9	208	6, 2.9%	18, 0.8%
	10	187	8, 4.9%	20, 1.0%
	11	203	18, 8.9%	57, 2.3%
	12	216	8, 3.7%	22, 1.0%
SP3 mean (SD) %	9–12	202 (12)*	10 (5), 5.1%	29 (19), 1.3%
Overall mean (SD) %	1–12	206 (41)	15 (13), 7.3%	48 (40), 2.2%

Speaking turns: SP1 vs. SP2 p value = 0.007, SP1 vs. SP3 p value = 0.006, SP 2 vs. SP3 p value = NS

Instances of overlapping speech and errors related to overlapping speech and medical terminology are shown in Table 5. Overlapping speech occurred an average of 17 ± 10 times per interaction. Most, though not all, instances of overlapping speech resulted in errors. Out of all total word errors (substitutions, deletions, and insertions) and mis-attributed speaker word errors, 16 ± 12 total word errors and 8 ± 7 mis-attributed speaker word errors were associated with overlapping speech, representing 19.1% of total word errors and 16.3% of mis-attributed speaker word errors. SP2’s interactions had the fewest instances of overlapping speech (5 ± 3), compared with SP1 (20 ± 9; *p* = 0.018) and SP3 (25 ± 4; *p* < 0.001). Medical terminology errors averaged 13 ± 5 incorrect words per interaction, accounting for 15% of total word errors. These medical terminology errors resulted in 8 ± 4 semantic word/phrase errors, representing 42% of all semantic word/phrase errors. Examples of mis-transcribed medical terminology errors include antacids, angina, myocardial infarction, gastroesophageal reflux disease, bruits, carotid artery, abdominal, esophageal, umbilical, hyperlipidemia, atorvastatin, lisinopril, echocardiogram, antiplatelet, troponin, and coronary artery disease. After observing these errors, we tried modifying the prompt to alert the LLM to expected medical terminology; however, this adjustment did not affect the LLM’s errors.

**Table 5:** Comparison of Errors associated with overlapping speech and medical terminology. Percent word errors and mis-attributed speaker word errors associated with overlapping speech and medical terminology errors are calculated relative to total word errors (deletions, substitutions, and insertions). Percent turn-taking errors associated with overlapping speech are calculated relative to all turn-taking errors

	Student	Overlapping speech instances	Word errors associated with overlapping speech	Turn-taking errors associated with overlapping speech	Mis-attributed speaker word errors associated with overlapping speech	Medical Terminology Errors
SP1	1	18	23, 18.1%	5, 10.6%	17, 12.1%	10, 7.9%
	2	14	17, 26.2%	2, 22.2%	5, 16.1%	14, 20.0%
	3	32	18, 16.8%	6, 26.1%	17, 29.3%	12, 11.2%
	4	14	11, 10.6%	4, 17.4%	8, 12.9%	15, 14.4%
SP1 mean (SD)%	1–4	20 (9)*	17 (5)17.1%	4 (2)16.7%	12 (6)16.1%	13 (2)12.7%
SP2	5	6	3, 3.9%	1, 14.3%	2, 4.7%	20, 26.0%
	6	5	3, 3.6%	2, 28.6%	4, 19.0%	20, 23.8%
	7	8	7, 16.7%	1, 4.5%	3, 2.9%	15, 35.7%
	8	1	0	1, 50.0%	1, 33.3%	13, 35.1%
SP2 mean (SD) %	5–8	5 (3)*	3 (3), 5.4%	1 (1), 13.2%	3 (1), 5.9%	17 (4), 28.3%
SP3	9	29	23, 30.3%	1, 16.7%	4, 22.2%	6, 7.9%
	10	22	23, 33.8%	1, 12.5%	8, 40.0%	6, 8.8%
	11	27	41, 43.6%	5, 27.8%	21, 36.8%	15, 16.0%
	12	22	19, 18.6%	2, 25.0%	4, 18.2%	5, 4.9%
SP3, mean (SD) %	9–12	25 (4)*	27 (10), 31.2%	2 (2), 22.5%	9 (8), 31.6%	8 (5), 9.4%
Overall mean SD) %	1–12	17 (10)	16 (12) 19.1%	3 (2), 17.2%	8 (7), 16.3%	13 (5), 15.4%

Overlapping speech segments: SP1 vs. SP 2 t test p value 0.018, SP1 vs. SP3 p value NS, SP2 vs. SP3 p value < 0.001

We evaluated the associations between target word counts, speaking turns, overlapping speech, and error patterns. In exploratory analyses, several moderate associations were observed. The number of total target words correlated moderately with speaking turns (*r* = 0.55) and overlapping speech (*r* = 0.31). Speaking turns correlated moderately with total word errors (*r* = 0.41), turn-taking errors (*r* = 0.62), mis-attributed speaker word errors (*r* = 0.51), word errors associated with overlapping speech (*r* = 0.45), turn-taking errors associated with overlapping speech (*r* = 0.59), and mis-attributed speaker word errors associated with overlapping speech (overall *r* = 0.51). Overlapping speech correlated moderately with total word errors (*r* = 0.53), but only weakly with turn-taking errors (overall *r* = 0.23) and mis-attributed speaker word errors (overall *r* = 0.07).

Recordings obtained using higher-fidelity audio (HFA) (Supplemental Table 2) demonstrated significantly fewer total target word errors than original-fidelity audio (OFA) (23 ± 10, 1.0% vs. 71 ± 25, 3.2%, respectively, *p* = 0.001), primarily due to reduced deletions (8 ± 5, 0.4% vs. 56 ± 25, 2.5%, respectively, *p* = 0.001). Semantic word/phrase errors were also significantly lower in HFA recordings (10 ± 5 vs. 19 ± 5, *p* = 0.011). These improvements were consistent across both SP and student dialogue (Supplemental Table 3).

Turn-taking errors were reduced in HFA (4 ± 3, 1.9%) compared with OFA (11 ± 8, 5.3%) (Supplemental Table 4). Mis-attributed speaker word errors were lower in HFA (15 ± 13; 0.7%) than OFA (33 ± 23; 1.5%). Sematic errors related to turn taking were infrequent and significantly reduced in HFA (HFA: 1 ± 1 vs. OFA: 4 ± 2, *p* = 0.008).

When errors in the OFA were adjusted for the errors associated with overlapping speech and errors in HFA, 27 ± 25 total word errors—sum of substitutions, deletions, and insertions—were attributable to audio fidelity (1.2% of target words), and 19 ± 14 were attributable to overlapping speech (0.8%). Ten ± 20 mis-attributed speaker word errors (0.5%) were attributable to audio fidelity, and 8 ± 7 were attributable to overlapping speech (0.4%).

## Discussion

We evaluated the ability of a single LLM, NotebookLM, to transcribe speech between a “patient” (SP) and a “physician” (medical student). Overall, the LLM exhibited modest error rates, with an average target word error rate of 3.3% (range 2.3% to 4.1%) with 19 ± 5 semantic word/phrase errors per interaction. The most common error type was deletion, followed by substitution, and insertions were rare. Mis-attributed speaker word errors occurred on average in 2.2% of words (range 1.3% to 3.2%), with an average of 5 ± 6 semantic turn-taking errors.

Medical terminology errors were relatively common, accounting for approximately one of seven total word errors and three of seven semantic word/phrase errors. These errors involved medication names, medical conditions, symptoms, anatomical terms, physical examination findings, and laboratory or diagnostic tests. Medical terminology errors can lead to delays, inefficiencies in care, and patient harm [20–22]. Notably, prompting NotebookLM to anticipate specific terminology did not improve performance, underscoring limitations in its handling of medical vocabulary.

Though all interactions had modest error rates, there was variability in error rates amongst individual SP-student interactions. Conversation structure strongly influenced accuracy. The number of target words was moderately associated with speaking turns and overlapping speech. Speaking turns were moderately associated with total word errors, turn taking errors and mis-attributed speaker word errors, and errors associated with overlapping speech. Overlapping speech was moderately associated with total word errors but weakly associated with turn-taking errors and mis-attributed speaker word errors. Across participants, SP1’s interactions contained the highest number of speaking turns, target word errors, and mis-attribution errors, whereas SP2’s dialogue had the least speaking turns, instances of overlapping speech, and the target word errors. These findings suggest that clear, orderly turn-taking improved LLM accuracy.

Higher-fidelity audio (HFA) significantly improved transcription accuracy. Both target word errors and semantic errors were reduced; there was a large decrease in deletions. These improvements are likely due to both enhanced audio quality and reduced overlapping speech in the re-enacted encounters, although the overlap-adjusted analysis suggests that audio fidelity accounted for the majority of the reduction in errors. Even under ideal conditions, persistent semantic errors remained, which impaired accurate clinical transcription.

We identified overlapping speech, turn-taking, medical terminology, and lower audio fidelity were frequent causes of errors in the NotebookLM-generated transcriptions, but many errors occurred without a discernible cause. Remedies for some of the errors include using LLMs trained on medical terminology [23], high quality audio, reducing overlapping speech, and clear turn-taking. However, achieving these conditions consistently in real-world clinical settings may be difficult. As a result, transcription errors are likely to persist, and even modest errors can have a meaningful impact on clinical documentation.

Our study has several limitations. The pilot comparison was limited to two interactions and was not designed to support broad claims about relative performance across all LLM transcription systems; rather, a single platform was selected to ensure internal consistency within this study. Transcription accuracy may differ across LLMs, and future work should include multi-scenario benchmarking across several LLM-based transcription tools. In addition, the evaluation only included three standardized patients and 12 total interactions. While this yielded sufficient text volume for detailed analyses, the controlled setting and limited clinical diversity may restrict generalizability to other patient complaints, patient demographics, clinical environments, and varying levels of overlapping speech or background noise. Strengths include the use of a single high-quality clinical scenario, trained SPs, and a controlled environment, all of which enhanced reliability and reduced variability within and across encounters.

LLMs are currently being used for autonomous transcription, which becomes the basis for their clinical notes. Because ambiently-generated notes deliberately summarize and filter what they perceive as irrelevant, important social, contextual, and relational information may be lost [24, 25]. Our study found modest error rates, yet meaningful errors persisted that could undermine documentation accuracy, confuse clinicians, and cause patient harm. We suggest that medicine is not yet ready for widespread autonomous LLM note generation [26].

There are two primary ways to incorporate LLMs into clinical note generation and review. In one approach, physicians author the note and the LLM assists in reviewing and refining it. In the other, the LLM generates the note and the physician reviews and corrects it. Prior studies show that physicians do not reliably detect or correct all transcription errors [27, 28]. Over time, reviewing large numbers of autonomously generated notes may contribute to fatigue, incomplete review, or overlooked inaccuracies. A potentially critical issue with the physician reviewing the LLM’s clinical note is skill decay. Traditional documentation requires clinicians to acquire and synthesize medical information and create problem lists, differential diagnoses, and management plans [29, 30]. Reliance on LLMs for these cognitive tasks may diminish physicians’ diagnostic reasoning, foster skill decay, and erode accountability [31, 32]. This could adversely affect documentation quality and patient safety. If physicians disengage from clinical reasoning, no safeguard remains to verify or challenge an LLM’s output, which is a critical concern given these models’ susceptibility to error and bias [33–35]. Finally, if a patient is harmed due to an LLM error, who or what is responsible? Taken together, these considerations suggest that LLMs may be most appropriately used in a supportive role, such as assisting clinicians in reviewing, refining, and improving physician-authored notes, rather than replacing clinician participation in the documentation process.

## Conclusions

Accurate, efficient, and reliable physician notes are essential to safe, high-quality medical care [29, 36, 37]. Prior evidence on LLM transcription has been limited, heterogeneous, and rarely evaluated under standardized conditions. We systematically evaluated mis-transcription, mis-attribution, and medical terminology errors and the influence of audio quality in physician-patient interactions with a single LLM, NotebookLM, within a controlled simulated setting. Our study provides a benchmark for evaluating LLM transcription performance under simulated encounters that may inform the development, deployment, and evaluation of LLM in similar conditions.

Overlapping speech, turn-taking, medical terminology, and audio fidelity were frequent contributors to transcription errors with NotebookLM. Although overall LLM transcription errors were modest, even small numbers of errors may meaningfully affect documentation accuracy and interpretation. These findings suggest that caution is warranted when relying on LLMs for fully autonomous clinical note generation. LLMs may be most appropriately used in a supportive role, such as assisting clinicians in reviewing and improving physician-authored documentation, rather than replacing clinician involvement in the documentation process.

## Supplementary Information

Below is the link to the electronic supplementary material.

Supplementary Material 1

## Data Availability

Data used in this study consist of educational audio recordings and transcripts. Due to educational privacy restrictions, these data are not publicly available. All data relevant to the findings are included in this article, and additional details are available from the corresponding author upon reasonable request.

## Declarations

### Use of artificial intelligence

ChatGPT (GPT-5) was used to assist with editing and language refinement. The authors take responsibility for the integrity and accuracy of all content.

### Data responsibility

The first author had full access to all the data in the study and takes responsibility for the integrity of the data and the accuracy of the data analysis.

## Abbreviations

LLM: Large-language model
SP: Standardized patient
SD: Standard deviation
OFA: Original-fidelity audio
HFA: Higher-fidelity audio
