# Supplementary material for: Accuracy of large language model transcription of simulated physician-patient verbal interactions
Source: BMC Med Inform Decis Mak. 2026 Mar 10;26:124. doi: 10.1186/s12911-026-03414-3 (PMC13088782; doi:10.1186/s12911-026-03414-3)
Supplement: Supplementary file 1 — Supplementary Material 1 [file 12911_2026_3414_MOESM1_ESM.docx]

**Supplemental Table 1. Pilot Comparison of transcription errors by NotebookLM, AssemblyAI, and OtterAI across 2 interactions. Percentages are out of total target words.**

|  |  | Target words | Total word errors  (sum of substitutions, deletions, and insertions) | Substitutions | Deletions | Insertions | Misattributed speaker word errors |
| --- | --- | --- | --- | --- | --- | --- | --- |
| Notebook  -LM | Student 1  % | 2139 | 127  (5.9%) | 56  (2.6%) | 56  (2.6%) | 15  (0.7%) | 141  (6.6%) |
|  | Student 2  % | 2693 | 65  (2.4%) | 18  (0.7%) | 43  (1.6%) | 4  (0.1%) | 31  (1.2%) |
|  | Students 1-2 mean  % | 2416 | 96  (4.0%) | 37  (42.8%) | 50  (2.0%) | 10  (0.4%) | 86  (3.6%) |
| Assembly  -AI | Student 1  % | 2139 | 256  (12.0%) | 59  (2.8%) | 178  (8.3%) | 19  (0.9%) | 55  (2.6%) |
|  | Student 2  % | 2693 | 247  (9.2%) | 18  (0.7%) | 217  (8.1%) | 12  (0.4%) | 13  (0.5%) |
|  | Students 1-2 mean  % | 2416 | 252  (10.4%) | 39  (1.6%) | 198  (8.2%) | 16  (0.6%) | 34  (1.4%) |
| OtterAI | Student 1  % | 2139 | 452  (21.1%) | 84  (3.9%) | 347  (16.2%) | 21  (1.0%) | 188  (8.8%) |
|  | Student 2  % | 2693 | 443  (16.5%) | 47  (1.7%) | 384  (14.3%) | 12  (0.4%) | 243  (9.0%) |
|  | Students 1-2 mean  % | 2416 | 448  (18.5%) | 66  (2.7%) | 366  (15.1%) | 17  (0.7%) | 216  (8.9%) |

**Supplemental Table 2. Comparison of LLM total word errors in original-fidelity audio (OFA) and higher-fidelity audio (HFA). Percentages are out of total target words.**

|  | Interactions |  | Target Word Errors | | | Insertion errors | Sematic word/phrase errors  (may span multiple words) |
| --- | --- | --- | --- | --- | --- | --- | --- |
|  |  | Target words | Total target word errors | Substitution errors | Deletion errors |  |  |
| OFA  Mean (SD)  % | 1-6 | 2226 (303) | 71 (25) 3.2% | 15 (5)  0.7% | 56 (25)  2.5% | 8 (3)  0.3% | 19 (5) |
| HFA  Mean (SD)  % | 1-6 |  | 23 (10) 1.0% | 14 (6)  0.6% | 8 (5)  0.4% | 10 (1)  0.5% | 10 (5) |

Mean target word errors, p value 0.001; substitutions, p value NS, deletions, p value 0.001; insertions p value NS; semantic errors, p value 0.011.

**Supplemental Table 3 Comparison of LLM word errors by speaker in OFA and HFA. Percentages are out of total target words.**

|  | Standardized Patient (SP) Speaker | | | | | | Student Speaker | | | | | |
| --- | --- | --- | --- | --- | --- | --- | --- | --- | --- | --- | --- | --- |
|  | Target Words (SD) | Target Word Errors | | | Insertion errors | Sematic word/phrase errors  (may span multiple words) |  | Target Word Errors | | | Insertion errors | Sematic word/phrase errors  (may span multiple words) |
|  |  | Total target word errors | Substitu-  tion errors | Deletion errors |  |  | Target words | Total target word errors | Substitu-  tion errors | Deletion errors |  |  |
| OFA  Mean (SD) % | 583 (150) | 36 (20) 5.2% | 7 (3)  1.2% | 29 (19) 5.0% | 4 (2) 0.7% | 12 (4) | 1643 (170) | 35 (17) 2.1% | 8 (4)  0.5% | 27 (18) 1.6% | 4 (2)  0.2% | 7 (4) |
| HFA  Mean (SD) % |  | 11 (3) 1.9% | 6 (2)  1.0% | 5 (4)  0.9% | 4 (1) 0.7% | 4 (1) |  | 12 (8) 0.7% | 8 (6)  0.5% | 3 (2) 0.2% | 6 (2) 0.4% | 5 (5) |

SP data: target word errors, p value 0.013; substitutions, p value NS, deletions, p value 0.013; insertions p value NS; semantic errors, p value 0.001.

Student data: target word errors, p value 0.013; substitutions, p value NS; deletions p value 0.009; insertions p value NS; semantic errors, p value NS.

**Supplemental Table 4 Comparison of LLM turn-taking errors and misattributed speaker word errors in OFA and HFA. Percent of total turn-taking errors is out of speaking turns; percent of mis-attributed speaker word errors are out of total target words.**

|  | Interactions | Speaking Turns | Turn-taking errors | Misattributed speaker  word errors |
| --- | --- | --- | --- | --- |
| OFA  Mean (SD)  % | 1-6 | 208 (28) | 11 (8)  5.3% | 33 (23)  1.5% |
| HFA  Mean (SD)  % | 1-6 |  | 4 (3)  1.9% | 15 (13)  0.7% |

Turn-taking errors p value 0.073. Misattributed speaker word errors p value NS.
